# *My Name Is Legion, for We Are Many*—The Complex Community of Antibody Receptors

**DOI:** 10.3390/ijms242015226

**Published:** 2023-10-16

**Authors:** Markus Biburger

**Affiliations:** 1 Division of Genetics, Department of Biology, Friedrich-Alexander-Universität Erlangen-Nürnberg, 91058 Erlangen, Germany; markus.biburger@fau.de; 2 FAU Profile Center Immunomedicine (FAU I-MED), Friedrich-Alexander-Universität Erlangen-Nürnberg, 91054 Erlangen, Germany

Antibodies act as the central mediators of immunological defense mechanisms, therapeutic agents within clinics, and the mediators of various immune-mediated disorders. Whilst these immunoglobulins accomplish some of their biological functions autonomously, such as the neutralization of toxins, blocking the binding sites of receptors or ligands, etc., many of their other effector functions strictly depend upon interactions with distinct receptors (FcR) that bind antibody Fc domains.

Even the range of the names themselves—being numbered from α to μ, I to IV, and beyond—signifies the extensive variety of receptors that bind to antibodies. The purpose of this Special Issue on “Fc Receptors” is to report recent advances in the exceedingly complex topic of the interactions between these receptors and their antibody ligands, as well as the consequences of these interactions.

Due to the sheer complexity of the topic, as well as the overwhelming number of excellent primary research manuscripts and reviews that have been published in recent years, it would not be possible to acknowledge the vast majority of authors if only a few could be mentioned in such a short editorial, and much less to include those manuscripts representing the breadth of the field. Thus, the references presented herein are specifically limited to the work that is related to this Special Issue on the various groups of Fc receptors.

Prior to antigen encounters, naive B cells express IgM and IgD B cell receptors on their surface. The activation of T cell-independent and T cell-dependent B cell responses can eventuate in class-switch recombination (CSR). CSR is an intrachromosomal DNA rearrangement of the immunoglobulin heavy-chain (IgH) locus, by which the Cμ region encoding the constant heavy-chain segment of IgM is substituted with Cγ, Cα or Cɛ. Accordingly, the initial phases of primary immune responses are dominated by the secretion of IgM antibodies. Eventually, IgG, IgA or IgE antibody isotypes appear, secreted by activated B cells that have undergone class-switching. In subsequent responses to the same antigen, IgG is predominantly produced. The decision on whether recombination takes place within the IgH locus during CSR and, thus, which antibody isotype will be expressed by the affected B cell, is influenced by distinct cytokines.

Since initial IgM expression takes place prior to somatic hypermutation, the affinity of IgM antibodies tends to be rather low. In a way, this is compensated for by the pentamerization of IgM, which results in ten antigen-binding sites per functional unit. Due to the large size of IgM pentamers, this isotype is mainly found in blood and, to a lesser extent, in lymph. Their smaller size enables the other isotypes, IgG, IgE and IgA, to easily diffuse into tissues.

IgG is the predominant antibody isotype in blood and extracellular fluid; IgE is also present here, yet at very low levels. IgA is the principal isotype of Ig in secreted bodily fluids. Each of these Ig isotypes have distinct receptors that mediate their cell-dependent effector functions, namely FcμR for IgM, FcαR for IgA, FcεR for IgE and, accordingly, FcγR for IgG. Despite the pronounced homologies between Ig/FcR effector functions between mice and men, there are some distinct differences. For example, mice lack a functional IgA/FcαRI effector arm, since FcαRI is present as a pseudogene. However, some distinctions may also exist for the antibody/receptor pairs that are correspondingly found in both species. Within this Special Issue, this is exemplified in the work of Kubagawa and colleagues. The authors outline differences between human and mouse FcµR, including their cellular distribution and IgM ligand binding [1].

In fact, antibody–Fc receptor interactions are further complicated by the fact that there are several subclasses of certain Ig isotypes, as well as there being several Fc receptor subtypes. For IgA, there are two subtypes: IgA1 and IgA2. The most complex matrix of interactions exists for IgG with its four subtypes (IgG1–4), with IgG2 comprising subclasses 2a (or 2c in distinct mouse strains) and 2b. These IgG antibodies find binding partners with Fcγ receptors I, IIB, III and IV in mice, and I, IIA, IIB, IIC, IIIA and IIIB in humans. Each of these possible antibody–receptor interactions are characterized by individual affinities between the binding partners, which are further modulated by the respective size of the immune complexes, i.e., the variable number of antibodies opsonizing a single antigen, which engage the cell surface receptors. The glycosylation pattern of the Fc region of an antibody affects its conformation, and thereby its affinity to a given receptor. Over recent decades, many methodologies have been utilized to characterize these antibody–receptor interactions. In this Special Issue, Forest-Nault et al. review the use of surface plasmon resonance (SPR)-based optical biosensors, which offer the real-time and label-free analysis of protein interactions, and have contributed to the discovery and development of therapeutic monoclonal antibodies. Here, the authors detail the use of different SPR biosensing approaches for the characterization of IgG–FcγR interactions, and discuss the latest SPR-derived conclusions on the influence of N-glycosylation upon these interactions [2].

Fc receptors show distinctive expression patterns in terms of their presence and abundance on various immune effector cells in a steady state, which, again, can be affected by physiological changes, such as immune responses, pregnancy, hormonal changes, etc. The outcome of the binding of immune complexes to cells of a given population is influenced by the relative affinities of the respective IgG subclasses to these receptors, as well as by the numbers of activating and inhibitory FcγRs on the cells’ surface. Quantitative receptor assessments are important for predicting the outcome of cellular Fcγ receptors’ engagement by antibodies or immune complexes, for example based on mathematical models. This Special Issue includes a study by Vorsatz and Friedrich et al., focused on the quantitation of FcγRs on tissue-resident macrophages, a diverse group of highly specialized cells with important roles in antibody effector functions. The presented quantities of Fcγ receptors on macrophages of murine livers, lungs, kidneys, brains, skin and spleens revealed a pronounced heterogeneity in the expression patterns among different tissue macrophages, possibly indicating their specialized functions within their individual niches in different organ environments [3].

The above-mentioned Fcγ receptors belong to the Ig gene superfamily, and are often referred to as canonical Type-I Fc receptors. Most of these receptors are involved in the activation of immunological cell functions, whereas there is only one inhibitory receptor, FcγRIIB, that exists in both mice and men. The GPI-linked FcγRIIIB, which lacks an intracellular signaling domain, is a Type-I Fc receptor that is found in humans but not in mice. Also, FcαRI and the high-affinity IgE receptor FcεRI belong to the Type-I receptors. The latter is the topic of a review by Arthur and Cruse, where the authors describe the structure and function of this multimeric receptor complex, the roles and potential targeting of its β subunit in regulating mast cell function, FcεRI trafficking and signaling, and how the manipulation of its splicing could be employed as a therapeutic approach [4].

So-called Type-II Fc receptors comprise several C-type lectin receptors, including the low-affinity IgE receptor FcεRII (CD23, although the binding of IgE to CD23 is not via sugars) and murine SIGN-R1, which binds the 2,6-sialylated antibody Fc.

Antibody binding does not only take place on cell surfaces. Tripartite motif-containing protein 21—TRIM21—is an intracellular protein that binds IgG, IgA and IgM antibodies and initiates the degradation of antibody-bound proteins in the intracellular antibody-mediated proteolysis pathway. Its primary function is to eliminate antibody-decorated viruses that have evaded extracellular neutralization and have permeated the cell membrane.

The neonatal Fc receptor, FcRn, is an intriguing antibody receptor that promotes antibody transport rather than participating in cellular signaling. Under slightly acidic conditions, FcRn binds IgG and releases it at neutral pH. The receptor derives its name from its function in the syncytiotrophoblasts of human placentas. These cells take up maternal IgG from the mother’s blood through endocytosis. In acidic endosomal conditions, IgG binds to FcRn and undergoes transcytosis to reach the fetal site of the syncytiotrophoblast, or, in rodents, the cells of the yolk sac endoderm, due to their inverted yolk sac placenta. In the gastrointestinal tract, FcRn facilitates the uni-directional transcytosis of IgG through epithelial cells lining the gut lumen. Thus, passive immunization can also be achieved after birth with the maternal IgG that is present in breast milk during lactation. In humans, most humoral immune competency is achieved via IgG transfer prior to birth, with little maternal IgG uptake from breast milk. However, a prominent uptake of IgG in rats and mice occurs both before and after birth via FcRn-mediated transfer across the inverted yolk sac placenta and intestine, respectively. Two manuscripts in this Special Issue are dedicated to FcRn: Qi and Cao re-examine the hitherto elucidated biological and thermodynamic properties of FcRn, and discuss modeling and simulation studies that probe the quantitative relationship between the in vivo persistence of IgG and its in vitro FcRn binding [5], whereas Fieux et al. provide a systematic review of the utilization of FcRn as a transporter for the nasal delivery of biologics [6].

Also, for polymeric immunoglobulins—dimeric IgA and pentameric IgM—there is a distinct receptor with transportation function: the polymeric Ig receptor, pIgR. This receptor is expressed by the epithelial cells of the gastrointestinal and respiratory tracts and the skin, as well as on the glandular epithelial cells of mammary tissue and the liver. After its synthesis, pIgR is directed to the basolateral membrane of the epithelial cell. There, it is available for binding to its antibody ligands, which are secreted by plasma cells in the subepithelial space. The pIgR binds these polymeric antibodies via their joining chain (J chain), a small protein that is critical for the formation and stabilization of the dimeric and pentameric Ig structures. The pIgR/Ig complex is then taken up by clathrin-dependent endocytosis, and undergoes transcytosis to the cell’s apical surface. The part of pIgR that binds to the antibody undergoes endo-proteolytic cleavage. This cleaved extracellular portion of pIgR is referred to as the secretory component (SC). The SC remains bound to the respective IgA dimer or IgM pentamer when the antibody is released from the remaining membrane-associated parts of pIgR via the proteolytic cleavage. The released SC/antibody complex is designated as secretory Ig. In their contribution to this Special Issue, Wei and Wang review pIgR’s role in IgA and IgM transcytosis, while also examining the roles of the J chain in the formation of polymeric IgA and IgM as well as in the recognition of these antibodies by pIgR. Moreover, they highlight recent advancements in the understanding of the molecular chaperone, MZB1 [7].

Given that antibodies have become essential in many therapeutic approaches, it is evident that their receptors are of clinical importance as well.

In this Issue, Gogesch, Dudek and colleagues review the impact of Fc receptors on the efficacy of therapeutic monoclonal antibodies. The authors describe Fc-mediated effector functions as being characteristic features of respective monoclonal antibodies, discuss safety aspects, describe methods for analyzing Fc–FcγR interactions, and discuss Fc–FcγR interaction-mediated side effects and novel strategies for the development of therapeutic antibodies, e.g., through antibody engineering in order to modify receptor interactions [8].

Three articles of this Special Issue deal with specific diseases or treatment regimens: Reviewing the current status of Fc receptor-targeted therapies for treating the autoimmune disease myasthenia gravis, Keller et al. illustrate the mechanisms of action and clinical efficacies of emerging Fc-mediated therapeutics, such as neonatal Fc receptor (FcRn)-targeting agents. Additionally, they assess the potential of therapies that target classical Fc receptors, which have already exhibited promising therapeutic efficacy in other antibody-mediated conditions [9].

In the human population, various polymorphisms of Fcγ receptors exist, which can cause differences in receptor expression or their affinity to their IgG ligands. Using a pharmacokinetics analysis of the therapeutic antibody infliximab (IgG1) in patients with controlled Crohn’s disease, Thibault and colleagues investigated its elimination in association with the patients’ platelet count and respective FcγRIIA polymorphisms. The authors propose that the elimination of infliximab, and possibly any IgG1 antibody, is reliant, in part, on binding to platelet FcγRIIA, a mechanism that has not been fully acknowledged [10].

Legrain et al. analyzed the role of FcγRs and Type I and Type II interferons in a mouse model of autoimmune anemia, caused by infection with the lactate dehydrogenase-elevating virus (LDV), via the enhanced macrophage-mediated phagocytosis of autoantibody-opsonized erythrocytes. The authors suggest that the interferon-mediated regulation of FcγRI and IV expression, but not FcγRIII expression, may contribute to the exacerbation of autoimmune anemia due to LDV infection [11].

In summary, this Special Issue on Fc receptors provides both recent research findings and comprehensive reviews on various aspects of antibody–Fc receptor interactions, ranging from individual Fc receptors to global aspects of the role of Fc receptors in monoclonal antibody therapies.

Due to the exceptional complexity of the interplay between antibodies and their receptors, and because of their significant clinical implications, a large body of compelling publications on this topic is anticipated in the coming years.
